# Physical Activity Habits and Sleep Duration According to Gender: A Cross-Sectional Study of Elementary School Children

**DOI:** 10.3390/healthcare12141400

**Published:** 2024-07-13

**Authors:** Josune Rodríguez-Negro, Iñaki Llodio, Javier Yanci

**Affiliations:** 1 Faculty of Education and Sport, University of the Basque Country (UPV/EHU), 48940 Leioa, Spain; 2 AKTIBOki: Research Group in Physical Activity, Physical Exercise and Sport, Sports and Physical Exercise Research Group (GIKAFIT), Department of Physical Education and Sport, Faculty of Education and Sport, University of the Basque Country (UPV/EHU), 01006 Vitoria-Gasteiz, Spain; inaki.llodio@ehu.eus (I.L.); javier.yanci@ehu.eus (J.Y.)

**Keywords:** child health, healthy habits, primary education, physical exercise, PAQ-C, childhood

## Abstract

(1) Background: The main goals of this study were to describe the physical activity (PA) and sleep habits of 8–12-year-old children according to their gender and to evaluate the relationship between PA and sleep habits (i.e., duration and timing). (2) Methods: A total of 236 children (114 boys and 122 girls) completed the Physical Activity Questionnaire for Older Children (PAQ-C) and an ad hoc sleep habits questionnaire. (3) Results: Boys were more physically active than girls (2.62 ± 0.51 vs. 2.46 ± 0.48, *p* = 0.026) and enacted higher PA levels in school recess (3.82 ± 1.36 vs. 3.56 ± 1.38, *p* = 0.003), during the afternoon (3.37 ± 1.20 vs. 2.89 ± 1.12, *p* = 0.003), and during weekends (3.54 ± 1.20 vs. 3.18 ± 0.48, *p* = 0.009). Per sleep habits, boys had a significantly later bedtime (21:53 ± 2:08 vs. 21:34 ± 2:14, *p* = 0.009) and a significantly smaller total sleep duration (9.64 ± 0.86 vs. 9.89 ± 0.87 h, *p* = 0.023) than girls. No significant correlations between PA and sleep habits were found. (4) Conclusions: We found differences in the PA and sleep habits between school-age boys and girls. Institutions and entities should consider designing specific interventions to improve PA and sleep habits according to gender.

## 1. Introduction

Healthy habits during childhood, like regular physical activity (PA), reduced sedentary behaviour, sufficient sleep, adequate nutrition, and limited screen time, are crucial for health [1,2,3]. Furthermore, this is of particular importance given that health during childhood is a strong predictor of health in adulthood; indeed, establishing positive habits in childhood is linked to improved lifelong health [4]. Unfortunately, there is a growing body of literature that has reported that most children around the world do not meet healthy habit recommendations [5,6,7]. 

According to international recommendations, children should accumulate at least 60 min of daily moderate-to-vigorous PA [8]. In recent decades, more emphasis has been placed on the effects of PA, and regular PA has been demonstrated to be one of the most appropriate ways to enhance numerous positive health outcomes in children, such as overweight/obesity reduction [2] and cardiovascular and metabolic chronic disease risk reduction [9]. Moreover, PA has been widely recognized for its benefits on psychological well-being, cognitive functions, and academic outcomes [10,11,12]. Despite the benefits of regular PA in children, two-thirds of children do not meet the international recommendations, and the lack of regular PA and increased time of sedentary behaviour are the fourth highest risk factor attributed to mortality globally [8,13,14]. In a study carried out with 49,606 U.S. children, Friel et al. [5] found that only 23% met the PA recommendations. Therefore, due to the importance and the lack of PA during childhood, more studies in different contexts are needed.

It is also widely accepted that sleep has restorative effects—a key component for children’s health and well-being [15]. Sleep has a wide range of functions, which include promoting development, improving working memory and cognitive functions, regulating behaviour and emotions, strengthening the immune system, and developing adequate mental health in school-age children [3,16,17]. Furthermore, it has been demonstrated that sleep duration is predictive of lower odds of obesity [18]. A substantial body of literature suggests that a lack of adequate sleep habits in children is associated with many adverse consequences, including physical, neurocognitive, emotional, and behavioural outcomes [3,19]. Even for children between 6 and 12 years old encouraged to sleep 9–12 h/day [20], insufficient sleep has become more common among children. While few studies have reported an average sleep duration between 9 and 11 h/night [21,22], many others have reported an average sleep duration under 8 h/night [23,24,25]. In a study carried out with 497 children, Lima et al. [23] found that only 26.1% of the Brazilian children and 33.7% of children from the west of Spain slept more than 8 h/day.

Given the importance of healthy habits during childhood, a myriad of studies have focused on whether school children are following health habit recommendations. However, these studies have analysed girls and boys as a whole group and, therefore, less is known about the differences of habits according to gender [24]. Some previous studies have found differences between boys and girls in some health habits like nutrition or screen time [24,25]. Moreover, it seems that there might also be gender differences in PA levels and sleep time [26,27]. While this information might be important for planning adequate health promotion interventions to foster healthy habits among children, the influence of gender in healthy habits during childhood has not been deeply explored yet. 

Moreover, it is important to understand both whether school-age boys and girls are getting enough PA and sleep and the relationship between these two variables. Previous studies found higher PA levels associated with improved sleep [19,28]. Kalak et al. [29] found that adolescents who ran 30 min/week had better sleep outcomes (i.e., increased slow-wave sleep, decreased sleep latency, and enhanced perceived sleep quality), although few researchers have focused on the relationship between PA and sleep in children. Therefore, even if current prevention and intervention approaches to address sleep problems usually include PA [19], it might be necessary to delve more deeply into this relationship, especially in children. Therefore, the main goals of this study were to describe the PA and sleep habits of 8–12-year-old children according to their gender and to evaluate the relationship between PA and their sleep habits.

## 2. Materials and Methods

### 2.1. Participants

A total of 258 children between 8 and 12 years old from a Spanish elementary education state school located in the city of Bilbao were invited to take part in this research, whereas a final 236 children (114 boys and 122 girls) took part in the study. The school was located in the Indautxu area of Bilbao. While in a state school the socioeconomic characteristics are varied, in general the sample was composed of middle–high socioeconomic class families, with an average family income of €76,661.

Table 1 shows the age, height, body mass, and body mass index (BMI) of all participants. The inclusion criteria were (1) not being sick the previous week or having something that precluded the child from doing their regular PA, and (2) completing both the PA and sleep habits questionnaires. Before participation, the children and parents or legal guardians were informed about the aim and the design of the study, and the parents or legal guardians signed an informed consent form. The management team of the elementary school to which the children belonged also approved the study. The study was performed in accordance with the Helsinki Declaration (2013) and was approved by the Ethics Committee (CEISH, code 2015/147) of the University of the Basque Country (UPV/EHU).

### 2.2. Procedure

In this study, we examined the PA and sleep habits of 8–12-year-old boys and girls. Both questionnaires were administered in paper and at the beginning of the second trimester of a school year. To assess PA habits, the Physical Activity Questionnaire for Older Children (PAQ-C) [30] was completed by participants during physical education class during school hours. A sleep habits questionnaire was sent to the families through the school and was completed by parents/guardians [31]. The information from both questionnaires was subsequently transferred to an Excel file.

### 2.3. Measures

The PAQ-C [30] was used to assess the PA habits of the participants. The PAQ-C is a self-administered 7-day recall questionnaire for children 8–14 years old. The questionnaire provides a summary of PA calculated from 10 items; the first nine items are scored on a 5-point scale (with 1 representing the lowest level and 5 representing the highest level of PA), while the last item is scored on a 2-point scale. The first item is a checklist of activities and common sports and games. The final six items assess PA during specific moments of the previous 7 days: physical education sessions, school recess, lunch time, afternoon (14–18 h), evening (18–22 h), and during the weekend. The eighth item evaluates the intensity of the PA, and the ninth item asks about the frequency of PA over each day of the previous week. Out of the total score calculation, item 10 asks children if they have been sick or if something prevented them from doing their regular PA. The average score on the questionnaire allows investigators to know the level of PA carried out during the last week by the children [32]. The PAQ-C had already been used in school-age children [33,34] and was found to correlate with moderate-to-vigorous physical activity (MVPA) scores measured with accelerometers [35]. Furthermore, the Spanish version of the PAQ-C has been found to have good reliability values (Cronbach’s alpha = 0.83) [36]. 

To analyse participants sleep habits, each child’s sleep timing (hour of bedtime and waketime) was parent/guardian-reported. Parents/guardians were asked: “At what time does your child usually fall asleep?” and “At what time does your child usually wake up in the morning?” From these data, the total sleep duration (hours/day) of each child was also calculated. Sleep duration and timing were similarly recorded in other studies with school-age children [1,5].

### 2.4. Statistical Analysis

The results are presented as means, standard deviations (SDs), frequencies, and percentages. To determine the normality of the data and the equality of variances, the Kolmogorov–Smirnov statistic and Levene’s test were used respectively. The Mann–Whitney U-test was used to determine differences between the girls and boys. The magnitudes of the differences were calculated using the probability of superiority (PS) [37]. The following qualitative interpretation was used to interpret the PS values: trivial (PS = 0.00–0.50), small (PS = 0.50–0.56), moderate (PS = 0.56–0.71), and large (PS > 0.71) differences between means [38]. Furthermore, the mean differences between the boys and girls were calculated in percentages (Dif. %): ([Mean girls − Mean boys] × 100)/Mean boys. Finally, Chi-square statistical analysis was used to analyse the statistical significance in the distribution of percentages of the answers for each group. Data analysis was performed using SPSS (Version 23) for Windows (Chicago, IL, USA). Statistical significance was set at *p* < 0.05.

## 3. Results

Table 2 shows the PA results on the PAQ-C for all the participants. The PAQ-C total score for all participants was 2.54 ± 0.50 points. The most-practiced PA were walking, jogging or running, football, and basketball, while the least-practiced were canoeing, badminton, and aerobics. The children were most active during physical education lessons and school recess.

Table 3 shows the PA results on the PAQ-C for the boys and girls and the differences according to gender. The boys achieved significantly higher results than the girls per the PAQ-C total score (2.62 ± 0.51 vs. 2.46 ± 0.48, *p* = 0.026, PS = 0.42, trivial). Regarding the practice of different sports (items 1A–1O), boys reported significantly higher values for football (3.54 ± 1.52 vs. 1.90 ± 1.16, *p* < 0.001, PS = 0.22, trivial; chi2 = 65.398, *p* < 0.001), in-line skating (1.56 ± 1.13 vs. 1.84 ± 1.09, *p* = 0.001, PS = 0.59, moderate; chi2 = 18.939, *p* = 0.001), skateboarding (1.83 ± 1.23 vs. 1.31 ± 0.72, *p* < 0.001, PS = 0.38, trivial; chi2 = 17.221, *p* = 0.002), cycling (2.25 ± 1.32 vs. 1.67 ± 1.05, *p* < 0.001, PS = 0.37, trivial; chi2 = 14.746, *p* = 0.005), and jogging or running (4.17 ± 1.27 vs. 3.70 ± 1.38, *p* = 0.005, PS = 0.40, trivial; chi2 = 9.794, *p* = 0.044). However, the girls reported higher values than the boys for skipping (1.27 ± 0.65 vs. 1.49 ± 0.73, *p* = 0.003, PS = 0.59, moderate; chi2 = 11.006, *p* = 0.026) and dance (1.36 ± 0.74 vs. 2.25 ± 1.34, *p* = 0.000, PS = 0.70, moderate; chi2 = 37.648, *p* < 0.001). Regarding PA during different moments of the day or the week, boys obtained higher values than girls on item 3 (PA during school recess; 3.82 ± 1.36 vs. 3.56 ± 1.38, *p* = 0.003, PS = 0.44, trivial; chi2 = 13.565, *p* = 0.009), item 5 (PA during the afternoon from 14:00 to 18:00 h) (3.37 ± 1.20 vs. 2.89 ± 1.12, *p* = 0.003, PS = 0.39, trivial; chi2 = 14.372, *p* = 0.006), and item 9F (PA on Saturdays) (3.88 ± 1.32 vs. 3.33 ± 1.35, *p* = 0.001, PS = 0.38, trivial; chi2 = 15.603, *p* = 0.004).

**Figure 1:**
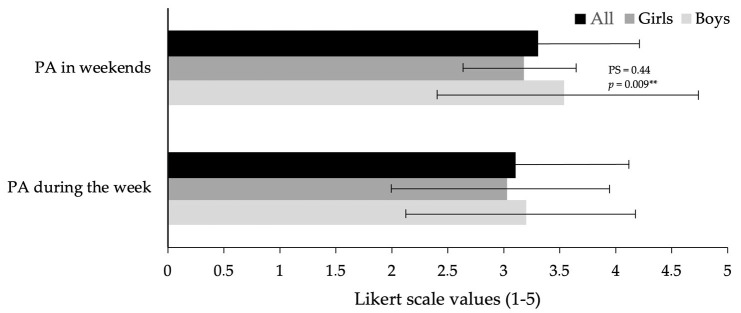
Week and weekend PA results for all participants and for boys and girls. Note: PA = physical activity. ** *p* < 0.01 = significant differences between boys and girls.

With item 9 (PA during each day of the week), the PA level during the week and weekend were calculated. The results for all participants were 3.11 ± 0.93 during the week and 3.36 ± 1.33 during the weekend. Boys achieved significantly higher PA results than girls during the weekends (3.54 ± 1.20 vs. 3.18 ± 0.48, *p* = 0.009, PS = 0.44, trivial). No differences were found during the week (3.20 ± 0.98 vs. 3.03 ± 0.88, *p* = 0.113, PS = 0.40, trivial) (Figure 1).

**Figure 2:**
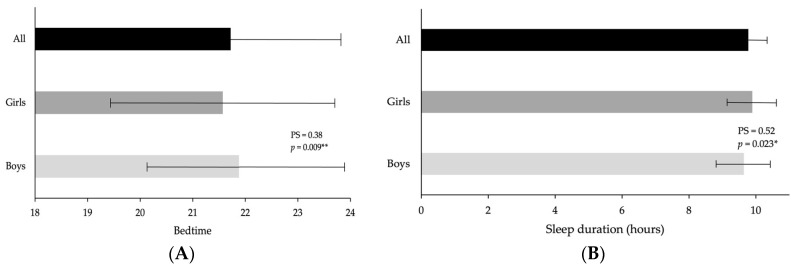
Sleep habits of the participants according to gender; (**A**) bedtime and (**B**) sleep duration. Note: * *p* < 0.05, ** *p* < 0.01 = significant differences between boys and girls.

According to sleep habits, participants’ bedtime was at 21:43 ± 2:11, and waketime at 7:44 ± 0:28, with a total sleep duration of 9.77 ± 0.88 h. No significant differences were found for gender (i.e., boys and girls) per waketime (7:43 ± 0:29 vs. 7:46 ± 0:26, *p* = 0.381, PS = 0.48, trivial). Still, the boys had a significantly later bedtime than the girls (21:53 ± 2:08 vs. 21:34 ± 2:14, *p* = 0.009, PS = 0.38, trivial), and the boys had a significantly shorter total sleep duration than girls (9.64 ± 0.86 vs. 9.89 ± 0.87 h, *p* = 0.023, PS = 0.52, small) (Figure 2).

Finally, correlations between the PA and sleep items were calculated, and no significant correlations between the PAQ-C total score and any of the sleep habit items were found for all participants, and neither for the boys nor girls (r = 0.07 to 0.11, *p* > 0.05).

## 4. Discussion

The main goals of this study were to describe the PA and sleep habits of 8–12-year-old children according to their gender and to evaluate the relationship between PA and sleep habits. One of the main contributions of this research is the description of healthy habits and preferences according to the gender of the children, since the study focused on the differences between boys and girls. In addition, as far as we know, few studies have analysed health habit (i.e., PA and sleep) differences between boys and girls in school-age children. In general, the boys were more physically active than the girls, and they accumulated higher PA levels during school recess, during the afternoon, and during the weekends compared to the girls. Furthermore, the boys went to bed significantly later and slept fewer hours per total sleep duration per night than the girls. These results may help in designing specific interventions to improve PA and sleep habits according to gender.

Regular PA during childhood is associated with better health and development [9,12] as well as with better PA habits during adulthood [4]. The PA habits results of the present study showed a medium PA level of the children (2.54/5 points), meaning that some of them did not follow the PA international guidelines [8]. These results are in line with previous research carried out in other countries that had shown that between 23% and 33% of school-age children do not meet PA recommendations [5,8,13]. If we focus on the results obtained in other regions of Spain, Pano-Rodriquez et al. [26] also found a medium PA level (3.0/5 points) in a similar study carried out with 954 children in Catalonia. In the present study, the most-practiced PAs by children were walking, running, football, and basketball, while the least-practiced were canoeing, badminton, and aerobics. Our results are partially in line with the results reported by Barja-Fernández et al. [39] and Arce-Larroy et al. [40], who found that the most-practiced PAs by school-age children were walking, aerobics, cycling, and walking, and walking, running, and football, respectively. Moreover, when we focused on the moment the children achieved the most PA during the week, our results are in line with previous researchers who concluded that children were most active during physical education lessons and school recess [40]. 

Taking all of this into account, it is clear that there is a need to increase children’s PA levels. Furthermore, despite walking, running, football, and basketball being the most- practiced activities, it might be necessary to foster some other kinds of activities that have several benefits for children. For example, activities involving body expression have been shown to be beneficial for cognitive functions in children of this age [11]. Conversely, it could be interesting to articulate school interventions to increase PA during physical education lessons and school recess, as well as raising awareness among families about the benefits of PA to help children achieve the recommended PA levels. 

Even if previous researchers concluded that many children do not attain the recommended daily PA levels, studies that focused on differences according to gender are scarce [27]. In this study, the overall results of our gender analysis showed significant differences between boys and girls in PA levels, with boys scoring higher than girls. Likewise, boys accumulated higher PA levels during school recess, during the afternoon, and during the weekends. Our results are in line with previous studies that suggested that primary school boys are more physically active than girls [26,27]. With a study conducted in Catalonia, Pano-Rodriguez et al. [26] also found significant differences between boys’ and girls’ total PA habits measured with the PAQ-C; the boys were significantly more active than the girls. The present study also showed that the boys practiced more in-line skating, skateboarding, jogging, running, cycling, and football than their counterparts, while the girls practiced significantly more skipping and dance than boys. Our results regarding PA preferences in children are partially in line with some previous results about PA preferences in adolescents, which showed that boys preferred team sports such as soccer, while adolescent girls showed preferences for inactivity, individual sports, or artistic activities like dance [41,42,43]. The differences in PA levels and preferred activities between boys and girls could be due to psychosocial factors [27]. Additionally, some researchers like Ávalos-Ramos et al. [41] have identified certain stereotyped patterns concerning sports selected based on gender. It may be necessary to modify those stereotypes and encourage boys and girls to practice more diverse activities, as varied PA practice can bring greater effects to children’s development and health [11]. Furthermore, taking into account that the girls seemed to practice less PA during the afternoons and weekends in this study, institutions and entities should consider policies that encourage girls to practice PA during these periods.

In addition to the performed PA levels, sleep habits have also been defined as important for children’s health [15,18]. The results of the present research showed a total sleep duration of 9.77 (±0.88) hours/night, with most children achieving the recommended daily hours of sleep. Previous studies had shown a lot of variability when analysing daily sleep duration in children and adolescents. Galland et al. [44], Grant and Gachupin [24], and Lima et al. [23] reported an average sleep duration under 8 h/night, while Whiting et al. [22] in a study with 150,651 children reported an average sleep duration between 9 and 11 h/night. These differences could be due to the age of the children or to the different cultural and environmental characteristics, as Spain seems to be one of the countries with better sleep habits among children [22]. Indeed, our results are in line with a previous study that concluded that >95% of Spanish children slept 9–11 h/night [22]. Again, the differences in sleep according to the children’s gender were also analysed. In the present study, the boys had a significantly later bedtime and significantly shorter total sleep duration per night than the girls. Even if some previous studies did not find a gender difference regarding sleep habits [22,24], some others, as well as the present study, found that girls slept longer than boys [45]. However, Pano-Rodriguez et al. [26], in a study carried out in Catalonia, did not find any differences in sleep duration between boys and girls, although they found differences in other sleep habit variables, like quality, latency, and disturbance. Therefore, it seems that sleep quality (as well as latency and disturbance) is generally better in boys than in girls [26,46]. Overall, females seem to be more vulnerable than males to sleep-specific stressors, which can contribute to girls’ increased risk for suffering from insomnia [47] or developing stress-related sleep disturbances throughout their lives [48]. Furthermore, due to biological and sociocultural factors that influence sleep patterns, sleep has generally appeared more impaired in girls [45]. These results highlight the need to investigate not only the duration of sleep but also its quality. Furthermore, due to the high variability of results, there is still controversy about the influence of gender on sleeping habits. Thus, it may be necessary to delve more deeply into the differences in sleeping habits in children per gender. 

Moreover, other studies have analysed the influence of PA on sleep habits [26,28]. According to the correlation between PA and sleep habits, in the present study, no significant correlations were found for all participants, and for neither the boys nor the girls. Previous studies had found an association between PA and sleep quality, sleep latency, wakefulness after sleep onset, nighttime awakenings, and sleep efficiency [26,49,50]. Furthermore, some studies had also concluded that regular PA had a positive impact on the time it took to fall asleep and sleep duration [28,51]. Afonso et al. [31] concluded that children who accumulated at least 60 min of daily PA went to bed earlier and slept longer. The causes behind the association between exercise and sleep found in other studies could be tied to the levels of fatigue after PA [52,53]. Even if current prevention and intervention approaches to address childhood sleep problems include PA [19], in the present study, no correlation between PA and sleep habits was found. In our study, most students had medium PA levels (2.54 ± 0.50 from 5 on the PAQ-C), so the lack of ‘very sedentary’ or ‘very active’ children might explain the lack of correlation between PA and sleep. Still, it would be interesting to continue research along these lines to understand the relationship between PA and sleep.

Although this research was carried out with adequate ethical and methodological rigor, this research had some notable limitations. Regarding the study sample, while it was not small (n = 236), it could have been larger. Also, this study used a convenience sample, and all the children were from the same primary school, so they represented a specific socioeconomic structure. On the other hand, the PA and sleep values were assessed through questionnaires and were not measured with accelerometers or other objective devices; future studies might measure these variables more objectively. Notwithstanding its limitations, this research had some strengths, likes its use of a rigorous methodology and child-validated questionnaire.

## 5. Conclusions

Overall, the PA levels of children in this study were medium (2.54/5 points) and should be increased. Moreover, the boys seemed to be more active than the girls, especially during afternoons and weekends, and different PA preferences according to gender were also found. Regarding sleep habits, sleep recommendations were generally followed, with a total sleep duration of 9.77 (±0.88) hours/night, although the boys went to bed significantly later and slept significantly fewer hours than the girls. These results could have important implications for families, schools, and health promotors since the results might be used to help foster healthy environments and inspire stakeholders to understand the need for planning interventions that promote PA among children, especially among school-age girls.

## Figures and Tables

**Table 1:** Participants’ physical characteristics.

	Age (Year)	Mass (kg)	Height (cm)	BMI (kg/m^2^)
All	9.66 ± 1.16	36.67 ± 9.08	139.68 ± 8.41	18.59 ± 3.14
Boys	9.75 ± 1.20	35.37 ± 7.44	139.32 ± 7.90	18.09 ± 2.53
Girls	9.57 ± 1.12	37.85 ± 10.24	140.01 ± 8.87	19.05 ± 3.55

BMI = body mass index.

**Table 2:** Physical activity results on the PAQ-C for all participants.

	Mean ± SD	1	2	3	4	5
Physical Activity in Your Spare Time: Have You Done Any of the Following Activities in the Past 7 Days? If Yes, How Many Times?		No	1–2 Times	3–4 Times	5–6 Times	7 Times or More
Item 1A: Skipping	1.39 ± 0.70	71.6% (169)	20.3% (48)	6.4 (15)	1.3% (3)	0.4% (1)
Item 1B: Rowing or canoeing	1.10 ± 0.42	93.6% (221)	3.8% (9)	2.1 (5)	0.0% (0)	0.4% (1)
Item 1C: In-line skating	1.71 ± 1.12	59.7% (141)	25.0% (59)	6.4% (15)	2.5% (6)	6.4% (15)
Item 1D: Skateboarding	1.56 ± 1.03	67.4% (159)	20.8% (49)	5.5% (13)	0.8% (2)	5.5% (13)
Item 1E: Walking	4.19 ± 1.29	7.2% (17)	7.2% (17)	11.0% (26)	8.9% (21)	65.7% (155)
Item 1F: Jogging or running	3.93 ± 1.35	7.6% (18)	11.0% (26)	16.1% (38)	11.4% (27)	53.8% (127)
Item 1G: Cycling	1.95 ± 1.22	49.2% (116)	27.1% (64)	11.4% (27)	4.2% (10)	8.1% (19)
Item 1H: Aerobics	1.21 ± 0.67	87.7% (207)	8.1% (19)	1.7% (4)	0.8% (2)	1.7% (4)
Item 1I: Dance	1.82 ± 1.18	55.5% (131)	25.0% (59)	7.6% (18)	5.5% (13)	6.4% (15)
Item 1J: Swimming	1.92 ± 1.19	50.4% (119)	25.0% (59)	11.4% (27)	7.6% (18)	5.5% (13)
Item 1K: Football	2.69 ± 1.57	33.5% (79)	19.1% (45)	16.5% (39)	6.8% (16)	24.2% (57)
Item 1L: Basketball	2.05 ± 1.12	39.8% (94)	30.9% (73)	19.1% (45)	4.7% (11)	5.5% (13)
Item 1M: Volleyball	1.26 ± 0.73	83.9% (198)	11.0% (26)	1.7% (4)	1.7% (4)	1.7% (4)
Item 1N: Badminton	1.14 ± 0.59	92.4% (218)	4.7% (11)	1.3% (3)	0.0% (0)	1.7% (4)
Item 1O: Other sport	1.94 ± 1.49	61.0% (144)	15.7% (37)	6.4% (15)	2.1% (5)	14.8% (35)
**In the last 7 days, during your PE classes, how often were you active?**		**I don’t do PE**	**Hardly ever**	**Sometimes**	**Quite often**	**Always**
Item 2: Physical education	3.76 ± 0.97	4.7% (11)	5.1% (12)	19.5% (46)	51.3% (121)	19.5% (46)
**In the last 7 days, what did you do most of the time at…**		**Sat down**	**Stood or walked around**	**Ran or played a little bit**	**Ran around and played quite a bit**	**Ran and played hard most of the time**
Item 3: School recess	3.68 ± 1.37	13.1% (31)	5.1% (12)	21.2% (50)	21.6% (51)	39.0% (92)
Item 4: Lunch	2.75 ± 1.45	30.9% (73)	11.9% (28)	25.8% (61)	14.4% (34)	16.9% (40)
**How many days in the last week did you do sports, dance, or play games in which you were very active during…**		**None**	**1 time last week**	**2 or 3 times last week**	**4 times last week**	**5 times last week**
Item 5: Afternoon (14–18 h)	3.12 ± 1.23	11.9% (28)	16.9% (40)	36.4% (86)	16.5% (39)	18.2% (43)
**How many days in the last week did you do sports, dance, or play games in which you were very active during…**		**None**	**1 time last week**	**2 or 3 times last week**	**4–5 times last week**	**6–7 times last week**
Item 6: Evening (18–22 h)	2.69 ± 1.19	19.5% (46)	23.3% (55)	35.6% (84)	12.3% (29)	9.3% (22)
Item 7: Weekend	3.36 ± 1.15	6.4% (15)	15.7% (37)	33.5% (79)	24.2% (57)	20.3% (48)
**Which one of the following describes you best for the last 7 days?**		**All or most of my free time was spent doing things that involved little physical effort**	**I sometimes (1–2 times last week) did physical things in my free time**	**I often (3–4 times last week) did physical things in my free time**	**I quite often (5–6 times last week) did physical things in my free time**	**I very often (7 or more times last week) did physical things in my free time**
Item 8: Intensity	3.11 ± 1.16	8.9% (21)	23.3% (55)	28.8% (68)	26.3% (62)	12.7% (30)
**How often did you do PA (like playing sports, games, dance or any other PA) for each day last week?**		**None**	**Little**	**Medium**	**Often**	**Very often**
Item 9A: Monday	2.91 ± 1.31	17.8% (42)	22.9% (54)	24.6% (58)	20.3% (48)	14.4% (34)
Item 9B: Tuesday	3.14 ± 1.36	14.8% (35)	20.3% (48)	22.0% (52)	21.2% (50)	21.6% (51)
Item 9C: Wednesday	2.97 ± 1.30	14.8% (35)	25.4% (60)	23.7% (56)	19.9% (47)	16.1% (38)
Item 9D: Thursday	3.04 ± 1.26	16.9% (40)	21.6% (51)	20.8% (49)	22.0% (52)	18.6% (44)
Item 9E: Friday	3.53 ± 1.43	13.6% (32)	13.6% (32)	15.3% (36)	21.2% (50)	36.4% (86)
Item 9F: Saturday	3.59 ± 1.36	10.6% (25)	12.7% (30)	19.5% (46)	21.2% (50)	36.0% (85)
Item 9G: Sunday	3.13 ± 1.87	19.1% (45)	19.9% (47)	19.5% (46)	19.1% (45)	22.4% (53)
PAQ-C total score	2.54 ± 0.50					

SD = standard deviation. Data of the Likert scale is shown in percentages (frequencies).

**Table 3:** Physical activity results on the PAQ-C for boys and girls and the differences according to gender.

	Boys	Girls	PS (Dif. %) and *p*	Boys					Girls					Chi2 *p*
	Mean ± SD	Mean ± SD		1	2	3	4	5	1	2	3	4	5	
**Physical Activity in Your Spare Time: Have you Done Any of the Following Activities in the Past 7 Days? If Yes, How Many Times?**				**No**	**1–2 Times**	**3–4 Times**	**5–6 Times**	**7 Times or More**	**No**	**1–2 Times**	**3–4 Times**	**5–6 Times**	**7 Times or More**	
Item 1A: Skipping	1.27 ± 0.65	1.49 ± 0.73	0.59 (17.32) *p* = 0.003 **	80.7% (92)	14.0% (16)	3.5% (4)	0.9% (1)	0.9% (1)	63.1% (77)	26.2% (32)	9.0% (11)	1.6% (2)	0.0% (0)	0.026 *
Item 1B: Rowing or canoeing	1.15 ± 0.55	1.05 ± 0.25	0.48 (−8.69) *p* = 0.133	91.2% (104)	4.4% (5)	3.5% (4)	0.0% (0)	0.9% (1)	95.9% (117)	3.3% (4)	0.8% (1)	0.0% (0)	0.0% (0)	0.333
Item 1C: In-line skating	1.56 ± 1.13	1.84 ± 1.09	0.59 (17.94) *p* = 0.001 **	72.8% (83)	14.0% (16)	4.4% (5)	1.8% (2)	7.0% (8)	47.5% (58)	35.2% (43)	8.2% (10)	3.3% (4)	5.7% (7)	0.001 **
Item 1D: Skateboarding	1.83 ± 1.23	1.31 ± 0.72	0.38 (−28.41) *p* < 0.001 **	55.3% (63)	26.3% (30)	7.9% (9)	0.9% (1)	9.6% (11)	78.7% (96)	15.6% (19)	3.3% (4)	0.8% (1)	1.6% (2)	0.002 **
Item 1E: Walking	4.17 ± 1.39	4.20 ± 1.19	0.49 (0.71) *p* = 0.687	10.5% (12)	6.1% (7)	7.9% (9)	7.0% (8)	68.4% (78)	4.1% (5)	8.2% (10)	13.9% (17)	10.7% (13)	63.1% (77)	0.146
Item 1F: Jogging or running	4.17 ± 1.27	3.70 ± 1.38	0.40 (−11.27) *p* = 0.005 **	7.0% (8)	6.1% (7)	13.2% (15)	10.5% (12)	63.2% (72)	8.2% (10)	15.6% (19)	18.9% (23)	12.3% (15)	45.1% (55)	0.044 *
Item 1G: Cycling	2.25 ± 1.32	1.67 ± 1.05	0.37 (−25.77) *p* < 0.001 **	37.7% (43)	28.9% (33)	15.8% (18)	6.1% (7)	11.4% (13)	59.8% (73)	25.4% (31)	7.4% (9)	2.5% (3)	4.9% (6)	0.005 **
Item 1H: Aerobics	1.19 ± 0.66	1.22 ± 0.68	0.51 (2.52) *p* = 0.680	88.6% (101)	7.9% (9)	0.9% (1)	0.9% (1)	1.8% (2)	86.9% (106)	8.2% (10)	2.5% (3)	0.8% (1)	1.6% (2)	0.924
Item 1I: Dance	1.36 ± 0.74	2.25 ± 1.34	0.70 (65.44) *p* < 0.001 **	73.7% (84)	21.1% (24)	2.6% (3)	0.9% (1)	1.8% (2)	38.5% (47)	28.7% (35)	12.3% (15)	9.8% (12)	10.7% (13)	0.000 **
Item 1J: Swimming	2.01 ± 1.20	1.85 ± 1.17	0.46 (−7.96) *p* = 0.200	45.6% (52)	27.2% (31)	14.9% (17)	5.3% (6)	7.0% (8)	54.9% (67)	23.0% (28)	8.2% (10)	9.8% (12)	4.1% (5)	0.179
Item 1K: Football	3.54 ± 1.52	1.90 ± 1.16	0.22 (−46.32) *p* < 0.001 **	15.8% (18)	12.3% (14)	17.5% (20)	11.4% (13)	43.0% (49)	50.0% (61)	25.4% (31)	15.6% (19)	2.5% (3)	6.6% (8)	0.000 **
Item 1L: Basketball	2.18 ± 1.22	1.93 ± 1.02	0.45 (−11.46) *p* = 0.186	36.8% (42)	30.7% (35)	18.4% (21)	6.1% (7)	7.9% (9)	42.6% (52)	31.1% (38)	19.7% (24)	3.3% (4)	3.3% (4)	0.425
Item 1M: Volleyball	1.25 ± 0.71	1.28 ± 0.75	0.51 (2.40) *p* = 0.650	85.1% (97)	9.6% (11)	2.6% (3)	0.9% (1)	1.8% (2)	82.8% (101)	12.3% (15)	0.8% (1)	2.5% (3)	1.6% (2)	0.658
Item 1N: Badminton	1.19 ± 0.71	1.09 ± 0.44	0.48 (−8.40) *p* = 0.247	90.4% (103)	5.3% (6)	1.8% (2)	0.0% (0)	2.6% (3)	94.3% (115)	4.1% (5)	0.8% (1)	0.0% (0)	0.8% (1)	0.612
Item 1O: Other sport	1.81 ± 1.35	2.07 ± 1.52	0.55 (14.36) *p* = 0.144	65.8% (75)	13.2% (15)	7.0% (8)	2.6% (3)	11.4% (13)	56.6% (69)	18.0% (22)	5.7% (7)	1.6% (2)	18.0 (22)	0.421
**In the last 7 days, during your PE classes, how often were you active?**				**I don´t do PE**	**Hardly ever**	**Sometimes**	**Quite often**	**Always**	**I don´t do PE**	**Hardly ever**	**Sometimes**	**Quite often**	**Always**	
Item 2: Physical education	3.74 ± 1.04	3.78 ± 0.92	0.50 (1.06) *p* = 0.941	6.1% (7)	4.4% (5)	20.2% (23)	48.2% (55)	21.1% (24)	3.3% (4)	5.7% (7)	18.9% (23)	54.1% (66)	18.0% (22)	0.741
**In the last 7 days, what did you do most of the time at…**				**Sat down**	**Stood or walked around**	**Ran or played a little bit**	**Ran around and played quite a bit**	**Ran and played hard most of the time**	**Sat down**	**Stood or walked around**	**Ran or played a little bit**	**Ran around and played quite a bit**	**Sat down**	
Item 3: School recess	3.82 ± 1.36	3.56 ± 1.38	0.44 (−6.80) *p* = 0.116	13.2% (15)	3.5% (4)	13.2% (15)	28.9% (33)	41.2% (47)	13.1% (16)	6.6% (8)	28.7% (35)	14.8% (18)	36.9% (45)	0.009 **
Item 4: Lunch	2.75 ± 1.47	2.74 ± 1.44	0.50 (−0.36) *p* = 0.933	32.5% (37)	9.6% (11)	24.6% (28)	16.7% (19)	16.7% (19)	29.5% (36)	13.9% (17)	27.0% (33)	12.3% (15)	17.2% (21)	0.734
**In the last 7 days, how many days did you do sports, dance, or play games in which you were very active during…**				**None**	**1 time last week**	**2 or 3 times last week**	**4 times last week**	**5 times last week**	**None**	**1 time last week**	**2 or 3 times last week**	**4 times last week**	**5 times last week**	
Item 5: Afternoon (14–18 h)	3.37 ± 1.30	2.89 ± 1.12	0.39 (−14.24) *p* = 0.003 **	11.4% (13)	11.4% (13)	33.3% (38)	16.7% (19)	27.2% (31)	12.3% (15)	22.1% (27)	39.3% (48)	16.4% (20)	9.8% (12)	0.006 **
**In the last 7 days, how many days did you do sports, dance, or play games in which you were very active during…**				**None**	**1 time last week**	**2 or 3 times last week**	**4–5 times last week**	**6–7 times last week**	**None**	**1 time last week**	**2 or 3 times last week**	**4–5 times last week**	**6–7 times last week**	
Item 6: Evening (18–22 h)	2.81 ± 1.09	1.26 ± 2.57	0.44 (−55.16) *p* = 0.074	14.0% (16)	21.1% (24)	43.0% (49)	14.0% (16)	7.9% (9)	24.6% (30)	25.4% (31)	28.7% (35)	10.7% (13)	10.7% (13)	0.082
Item 7: Weekend	3.52 ± 1.12	1.17 ± 3.22	0.43 (−66.76) *p* = 0.065	3.5% (4)	14.9% (17)	32.5% (37)	24.6% (28)	24.6% (28)	9.0% (11)	16.4% (20)	34.4% (42)	23.8% (29)	16.4% (20)	0.296
**Which one of the following describes you best for the last 7 days?**				**All or most of my free time did things with little physical effort**	**I sometimes (1–2 times last week) did physical things in my free time**	**I often (3–4 times last week) did physical things in my free time**	**I quite often (5–6 times last week) did physical things in my free time**	**I very often (7 or more times last week) did physical things in my free time**	**All or most of my free time did things with little physical effort**	**I sometimes (1–2 times last week) did physical things in my free time**	**I often (3–4 times last week) did physical things in my free time**	**I quite often (5–6 times last week) did physical things in my free time**	**I very often (7 or more times last week) did physical things in my free time**	
Item 8: Intensity	3.21 ± 1.23	3.01 ± 1.09	0.45 (−6.23) *p* = 0.179	9.6% (11)	20.2% (23)	27.2% (31)	25.4% (29)	17.5% (20)	8.2% (10)	26.2% (32)	30.3% (37)	27.0% (33)	8.2% (10)	0.251
**How often did you do PA (like playing sports, games, dance, or any other PA) for each day last week?**				**None**	**Little**	**Medium**	**Often**	**Very often**	**None**	**Little**	**Medium**	**Often**	**Very often**	
Item 9A: Monday	3.04 ± 1.28	2.79 ± 1.32	0.44 (−8.22) *p* = 0.133	15.8% (18)	17.5% (20)	29.8% (34)	21.1% (24)	15.8% (18)	19.7% (24)	27.9% (34)	19.7% (24)	19.7% (24)	13.1% (16)	0.194
Item 9B: Tuesday	3.30 ± 1.38	3.00 ± 1.33	0.44 (−9.09) *p* = 0.087	14.0% (16)	15.8% (18)	22.8% (26)	21.1% (24)	26.3% (30)	15.6% (19)	24.6% (30)	21.3% (26)	21.3% (26)	17.2% (21)	0.324
Item 9C: Wednesday	2.93 ± 1.32	3.01 ± 1.28	0.52 (2.73) *p* = 0.618	15.8% (18)	25.4% (29)	26.3% (30)	14.9% (17)	17.5% (20)	13.9% (17)	25.4% (31)	21.3% (26)	24.6% (30)	14.8% (18)	0.432
Item 9D: Thursday	3.15 ± 1.35	2.93 ± 1.37	0.45 (−6.98) *p* = 0.221	15.8% (18)	17.5% (20)	21.9% (25)	25.4% (29)	19.3% (22)	18.0% (22)	25.4% (31)	19.7% (24)	18.9% (23)	18.0% (22)	0.522
Item 9E: Friday	3.62 ± 1.49	3.45 ± 1.38	0.45 (−4.69) *p* = 0.213	14.9% (17)	11.4% (13)	13.2% (15)	17.5% (20)	43.0% (49)	12.3% (15)	15.6% (19)	17.2% (21)	24.6% (30)	30.3% (37)	0.226
Item 9F: Saturday	3.88 ± 1.32	3.33 ± 1.35	0.38 (−14.17) *p* = 0.001 **	7.0% (8)	12.3% (14)	14.9% (17)	17.5% (20)	48.2% (55)	13.9% (17)	13.1% (16)	23.8% (29)	24.6% (30)	24.6% (30)	0.004 **
Item 9G: Sunday	3.22 ± 1.46	3.05 ± 2.21	0.44 (−5.27) *p* = 0.102	15.8% (18)	21.9% (25)	14.9% (17)	19.3% (22)	28.1% (32)	22.1% (27)	18.0% (22)	23.8% (29)	18.9% (23)	17.2% (21)	0.124
PAQ-C total score	2.62 ± 0.51	2.46 ± 0.48	0.42 (−6.10) *p* = 0.026 *											

SD = standard deviation. PS = probability of superiority. Dif. % = mean differences. * *p* < 0.05, ** *p* < 0.01 = significant differences between boys and girls. Data of the Likert scale is shown in percentages (frequencies).

## Data Availability

The data are unavailable due to privacy or ethical restrictions.
